# Usefulness of simultaneous measurement of brain and muscle regional oxygen saturation in shock patients: A report of three cases

**DOI:** 10.1002/ccr3.4715

**Published:** 2021-08-25

**Authors:** Arisa Muratsu, Tomoya Hirose, Mitsuo Ohnishi, Jotaro Tachino, Shunichiro Nakao, Ryosuke Takegawa, Tomohiko Sakai, Tadahiko Shiozaki, Takeshi Shimazu

**Affiliations:** ^1^ Department of Traumatology and Acute Critical Medicine Osaka University Graduate School of Medicine Suita Japan; ^2^ Traumatology and Critical Care Medical Center National Hospital Organization Osaka National Hospital Osaka‐city Japan

**Keywords:** brain rSO_2_, muscle rSO_2_, near‐infrared spectroscopy, rSO_2_ monitor, shock

## Abstract

The regional oxygen saturation (rSO₂) values of brain and muscle tissues can be measured simultaneously even if blood pressure cannot be measured due to circulatory failure associated with shock and may continuously reflect the oxygen supply‐demand balance.

## Graphical abstract

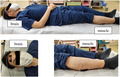

## 1 INTRODUCTION

We measured the regional oxygen saturation (rSO₂) values of brain and muscle simultaneously. Patients with shock showed that in the shock state, the value of crSO₂ was higher than that of mrSO₂, and c‐mDrSO₂ increased with the decrease of blood pressure. After resuscitation, the c‐mDrSO₂ decreased with the increase of blood pressure.

In the field of emergency medical care, we often experience a situation in which we cannot measure pulse oximetric saturation (SpO_2_) or blood pressure due to circulatory failure associated with shock. Regional oxygen saturation (rSO₂) is known as an indicator of changes in the local oxygen supply‐demand balance.^1^ Particularly, near‐infrared spectroscopy, which measures rSO₂ in the brain, can noninvasively measure the proportion of oxygenated hemoglobin at the site to be monitored in real time using the difference in the absorption of near‐infrared rays from oxygenated hemoglobin and reduced hemoglobin in the blood.^2^ This method can measure oxygen saturation in a non‐pulsatile flow environment and therefore can be used in patients with circulatory failure^3^ or after cardiac arrest.^4^ To our knowledge, however, no previous study has simultaneously evaluated the oxygen supply‐demand balance between central perfusion such as that in the brain and peripheral perfusion and that in muscle tissue in a noninvasive and real‐time manner in patients with shock.

We hypothesized that we could evaluate the oxygen supply‐demand balance between brain and muscle tissue by simultaneously measuring rSO₂ values of the brain and muscle tissues of patients in shock using an rSO₂ monitor (TOS‐OR; TOSTEC CO).^5^, ^6^ The study protocol was approved by the Institutional Review Board of Osaka University (approval no. 19540), which waived the need to obtain patient written informed consent because this was a noninvasive observational study.

Here, we show the rSO₂ values of the brain and muscle tissues of healthy volunteers and of three patients in shock in whom we preliminarily evaluated the rSO₂ values of the brain and muscle tissues during their resuscitation. We discuss the potential effectiveness of simultaneously assessing brain and muscle rSO₂ in shock management.

## 2 CASE PRESENTATIONS

### 2.1 Brain and muscle rSO_2_ in healthy volunteers

**FIGURE 1:**
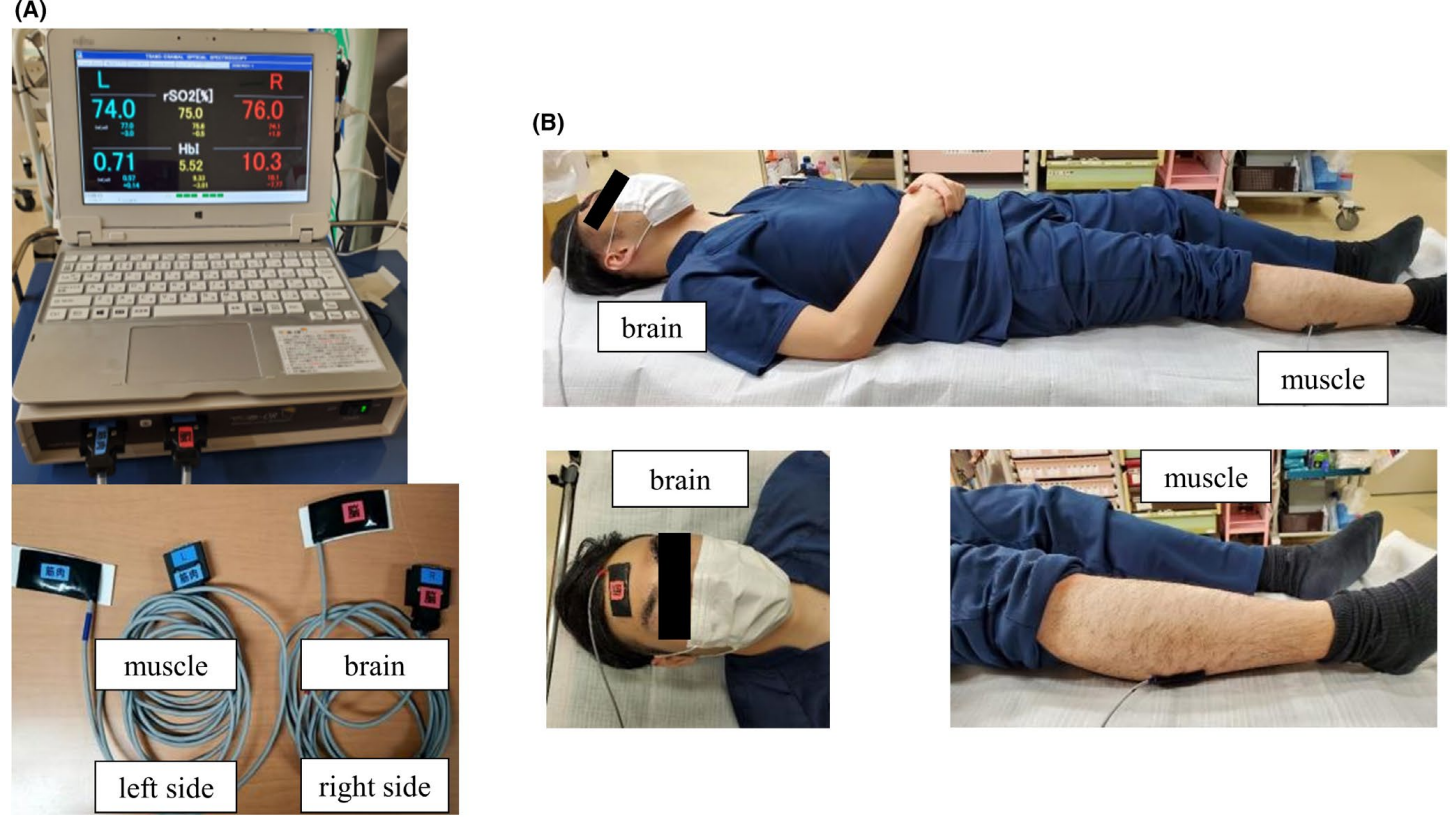
Photographs show the rSO₂ monitor and sensors and rSO₂ measurement in a mock patient. (A) The rSO₂ monitor (TOS‐OR; TOSTEC CO) can noninvasively measure the proportion of oxygenated hemoglobin in real time at the site to be monitored. This monitor has two sensors that are ordinarily attached to the left and right sides of the forehead. (B) In our study, we separated the sensors and attached one to the forehead and the other to the dorsal lower leg or dorsal upper arm to measure the rSO₂ values of the patient's brain and muscle, respectively. The sensors are shown attached to the forehead and dorsal lower leg of the mock patient

We attached the sensors of the rSO₂ monitor to the forehead and dorsal lower leg of 10 healthy volunteers lying in the supine position and measured rSO₂ values at these locations for 3 min (Figure 1). The reason for choosing the dorsal lower leg as the measurement site was that it was easy to attach the sensor when clothes are worn and measurement was less affected by body hair or physical constitution. When the sensor of the rSO₂ monitor was placed on the dorsal side of the lower leg, we confirmed by ultrasound that the depth from the skin surface to the muscle being measured by the sensor was 20–25 mm. Therefore, we thought the effects of body hair or physical constitution on rSO₂ were limited. The rSO₂ values of the volunteers' brain (cerebral regional oxygen saturation [crSO₂]) and muscle (muscle regional oxygen saturation [mrSO₂]) measurements were 77.6 ± 1.6% and 76.2 ± 1.3% (mean ± SD), respectively. There was little difference in cerebro‐muscular regional saturation of oxygen (c‐mDrSO₂) as indicated by the small difference between the crSO₂ and mrSO₂ values (Table 1).

**TABLE 1:** Values of cerebral regional oxygen saturation (crSO_2_) and muscle regional oxygen saturation (mrSO_2_) in healthy volunteers

Patient No.	Age (years)	Sex	sBP (mmHg)	dBP (mmHg)	MAP (mmHg)	crSO_2_ (%) mean SD		mrSO_2_ (%) mean SD	
1	29	M	121	84	96	78.6	0.3	74.5	0.3
2	28	M	150	100	117	81.3	0.5	76.2	0.5
3	43	M	156	115	129	79.2	0.4	76.9	0.4
4	29	M	109	63	78	76.3	1.2	76.3	1.3
5	39	M	124	80	95	77.7	0.8	76.9	0.7
6	33	F	109	75	86	77.9	0.7	77.0	0.7
7	32	F	107	71	83	75.1	0.4	77.1	0.4
8	29	M	102	70	81	77.2	0.8	78.1	1.1
9	34	M	153	82	106	77.0	0.9	73.4	1.0
10	32	M	117	74	88	77.0	0.3	75.3	0.2

Abbreviations: dBP, diastolic blood pressure; F, female; M, male; MAP, mean arterial pressure; No, number; sBP, systolic blood pressure; SD, standard deviation.

### 2.2 Shock case 1

**FIGURE 2:**
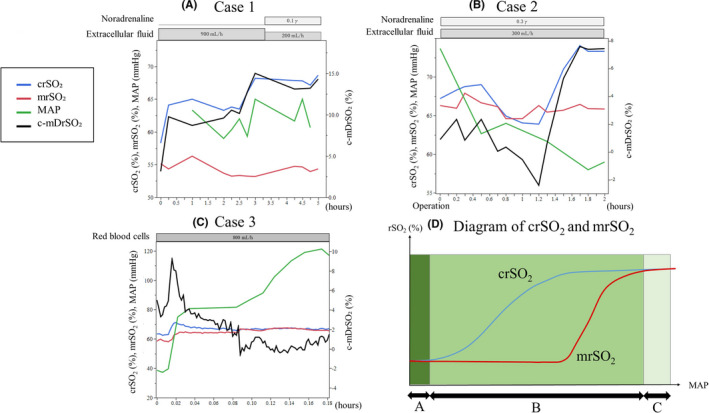
Serial changes of MAP, crSO₂, mrSO₂, and c‐mDrSO₂ in three patients with shock. (A) Case 1: Septic shock caused by bacterial pneumonia. (B) Case 2: Septic shock caused by bowel perforation. (C) Case 3: Hypovolemic shock caused by obstetric bleeding. D) A diagram of the expected crSO₂ and mrSO₂ waveforms. [C] In the case of stable MAP, the values of crSO₂ and mrSO₂ are almost equal and the c‐mDrSO₂ is small. [B] With a decreasing MAP, the value of mrSO₂ decreases and that of c‐mDrSO₂ increases. [A] In the case of remarkable hypovolemia, the values of both mrSO₂ and crSO₂ decrease, and the c‐mDrSO₂ also decreases. crSO_2_, cerebral regional oxygen saturation; mrSO_2_, muscle regional oxygen saturation; MAP, mean arterial pressure; c‐mDrSO₂, difference in cerebro‐musculoskeletal regional saturation of oxygen

A 56‐year‐old woman was admitted to our hospital with respiratory distress and malaise. On arrival, her heart rate was 145 beats per minute, and we could not palpate her radial artery pulse or measure SpO₂ because of her low blood pressure. We attached the sensors of the rSO₂ monitor to her forehead and dorsal lower leg and measured the rSO₂ values of her brain and muscle. Both values could be measured, and the value of crSO₂ was higher than that of mrSO₂. We diagnosed her condition as septic shock caused by bacterial pneumonia and immediately treated her with fluid resuscitation. Her mean arterial pressure (MAP) continued to be low, but after 1 h, we could measure a blood pressure by a noninvasive method. Meanwhile, the value of her crSO₂ remained higher than that of her mrSO₂. Although this patient's shock state was prolonged by septic shock caused by bacterial pneumonia, the value of her crSO₂ was always higher than that of mrSO₂, and the c‐mDrSO₂ was large (Figure 2A). She died on day 6 due to respiratory failure associated with pneumonia and bilateral pneumothorax.

### 2.3 Shock case 2

An 82‐year‐old man was transferred to our hospital with a diagnosis of septic shock caused by intestinal perforation. On admission, his blood pressure was 136/52 mmHg (MAP 73 mmHg), and his heart rate was 136 beats per minute under administration of noradrenaline and lactated ringers solution. We urgently performed partial resection of the intestine on the day of transfer. Just after the operation, his MAP was over 65 mmHg with the administration of noradrenaline, and his crSO₂ value was about the same as his mrSO₂ value. However, his MAP gradually decreased to below 60 mmHg at 1.6 h after the operation. Meanwhile, his crSO₂ value remained higher than that of his mrSO₂ value, and c‐mDrSO₂ increased with the decrease of the MAP. His MAP did not decrease any further by 1.8 h after the operation, and the c‐mDrSO₂ stabilized. This patient showed a decrease in his MAP with the development of septic shock caused by intestinal perforation. His crSO₂ value remained higher than that of his mrSO₂, and the c‐mDrSO₂ increased in tandem with the decrease of his MAP (Figure 2B). He was discharged on day 36.

### 2.4 Shock case 3

A 38‐year‐old woman was transferred to our hospital with a diagnosis of hemorrhagic shock following postpartum hemorrhage. On admission, her blood pressure was 74/40 mmHg (MAP 38 mmHg), and her heart rate was 86 beats per minute. We attached the rSO₂ sensor to her forehead and dorsal upper arm on admission and measured the values of crSO₂ and mrSO₂. The crSO₂ value was higher than that of her mrSO₂ value, and there was a c‐mDrSO₂. The bleeding from her uterus persisted, and her hemoglobin on admission was 6.5 g/dl. We began the administration of red blood cells at admission, and her MAP gradually increased. When her MAP rose above 85 mmHg, her crSO₂ value became about the same as that of her mrSO₂. In this patient with a low MAP due to hemorrhagic shock following postpartum hemorrhage, the value of her crSO₂ was higher than that of her mrSO₂ and a c‐mDrSO₂ was present. After her blood pressure began to increase, the c‐mDrSO₂ became much smaller (Figure 2C). After that, she received a hysterectomy and was discharged on day 11.

## 3 DISCUSSION

We revealed the following three points by simultaneously measuring the values of crSO₂ and mrSO₂. First, the value of crSO₂ is almost the same as that of mrSO₂ in healthy volunteers. Second, even if SpO₂ or blood pressure cannot be measured due to circulatory failure associated with shock, it is possible to measure crSO₂ and mrSO₂. Third, in the shock state, there is a difference in rSO₂ values between brain and muscle, and the value of crSO₂ is higher than that of mrSO₂. The present study is the first report, to our knowledge, to suggest that measurement of crSO₂ and mrSO₂ in shock patients may continuously and clearly reflect the oxygen supply‐demand balance.

There are three stages of shock: stage I (compensatory shock), stage II (decompensatory shock), and stage III (irreversible shock). Particularly, in the early phase of stage I, blood flow to the organs decreases, but blood flow in the major organs manages to be maintained by the physiological response generated to recover blood circulation.^7^ Previous studies assessed blood flow to the organs in shock patients by placement of an intravascular catheter^8^ or by pulse‐wave Doppler.^9^, ^10^ In contrast, we used the rSO₂ monitor as the tool to measure organ blood flow continuously and noninvasively.

Among the studies of patients in shock, there are reports that the value of crSO₂ is low when the MAP is low but that it increases with the increase in MAP.^3^, ^11^, ^12^, ^13^ Other reports on mrSO₂ showed a similar tendency to that of crSO₂.^14^, ^15^, ^16^

In this first report of the simultaneous measurement and assessment of the values of crSO₂ and mrSO₂ in shock patients, we illustrate in Figure 2D the expected change of crSO₂ and mrSO₂ as the MAP changes. In the case of a stable MAP, the values of crSO₂ and mrSO₂ are almost equal, and the c‐mDrSO₂ is small (C). In the case of decreased MAP, tissue blood flow to the non‐major organs decreases, and the value of mrSO₂ also decreases (B). For this reason, the value of crSO₂ is maintained at a higher value than that of rSO₂ (B), and c‐mDrSO₂ increases with the decrease in MAP. In the case of remarkable hypovolemia, blood flow to the brain decreases, and the value of crSO₂ also decreases. Thus, the values of crSO₂ and mrSO₂ are almost equal, and c‐mDrSO₂ decreases (A).

Case 1 shows that in patients with septic shock, MAP, crSO₂, and mrSO₂ change from (A) to (B) with the administration of fluid resuscitation and vasopressors. Case 2 shows that MAP, crSO₂, and mrSO₂ of the patients with septic shock change from (C) to (B). Case 3 shows that in patients with hypovolemic shock, MAP, crSO₂, and mrSO₂ change from (B) to (C) with the administration of red blood cells. The candidates for appropriately targeting MAP are those showing a change from (C) to (B) or from (A) to (B) to preserve major organ blood flow. We consider that stage I of the three stages of shock matches with (C), stage II matches with (B), and stage III matches with (A).

Our study showed that even if SpO₂ or blood pressure cannot be measured due to circulatory failure associated with shock, it is still possible to measure the values of crSO₂ and mrSO₂. Besides, because the values change in real time with fluctuation of the blood pressure, unlike with previous monitoring devices, the rSO₂ monitor may continuously and clearly reflect the changes in the local oxygen supply‐demand balance. Further, simultaneous measurement of crSO₂ and mrSO₂ rather than either crSO₂ or mrSO₂ alone may be helpful as a real‐time method for evaluating therapeutic effect.

A limitation of this study is the presence of selection bias associated with the choice of healthy volunteers and case reports used. We are continuing to accumulate additional cases and examining whether the simultaneous measurement of crSO₂ and mrSO₂ might be a useful method to evaluate adequate blood pressure in shock management. We will also consider the effects of therapeutic interventions such as the administration of noradrenalin or red blood cells.

## 4 CONCLUSION

We evaluated the usefulness of the simultaneous measurement of crSO₂ and mrSO₂ and found that it might be an objective and noninvasive method of evaluating blood pressure management in shock patients.

## CONFLICT OF INTEREST

None declared.

## AUTHOR CONTRIBUTIONS

A.M., R.T., and M.O. conceived the study and participated in its design. A.M., S.N., and J.T. collected and generated the data. A.M. wrote the first draft. T.H. and T. Shio. helped to draft the manuscript. A.M., T.H., R.T., M.O., S.N., J.T., T. Shio., T. Shima., and T. Sa. read and approved the final manuscript.

## ETHICAL APPROVAL

The study protocol was approved by the Institutional Review Board of Osaka University (Approval Number: 19540), which waived the need to obtain patient written informed consent because of the observational nature of the study.

## Data Availability

Not applicable.
